# Aortic Dissection With Cardiac Tamponade in Pregnancy: A Challenging Clinical Scenario

**DOI:** 10.7759/cureus.44126

**Published:** 2023-08-25

**Authors:** Daniela Barroso, Sérgio Santos, Ana Sofia Tomás, Heloísa Castro, António Pinheiro Vieira

**Affiliations:** 1 Internal Medicine, Centro Hospitalar Universitário de Santo António, Porto, PRT; 2 ACES (Agrupamento de Centros de Saúde) Grande Porto III-Maia/Valongo, Family Medicine, Maia, PRT; 3 Anesthesiology and Critical Care, Centro Hospitalar Universitário de Santo António, Porto, PRT; 4 Cardiology, Centro Hospitalar Universitário de Santo António, Porto, PRT

**Keywords:** case report, emergent perimortem cesarean delivery, pregnancy, cardiac tamponade, aortic dissection

## Abstract

Aortic dissection is the acute aortic syndrome with the highest mortality, and pregnancy and arterial hypertension are known risk factors. Its association with the perinatal period is a particularly unique and potentially devastating clinical catastrophe which is why the approach to a pregnant woman in cardiorespiratory arrest (CRA) should be multidisciplinary and early, with extraction of the fetus ideally within five minutes after the arrest. We present the case of a 39-year-old pregnant woman, who presented with a cardiorespiratory arrest in the context of an aortic dissection with cardiac tamponade and the need for an urgent perimortem cesarean section. Increasing knowledge and understanding among healthcare professionals has the potential to aid in the early detection and effective treatment of this challenging medical issue.

## Introduction

Aortic dissections are life-threatening complications that can arise from an underlying aortic aneurysm [1,2]. They occur when there is a tear in the innermost layer of the aortic wall, allowing blood to flow between the layers and potentially causing the aorta to rupture [2,3]. Aortic dissections are classified into two main types: Stanford type A, which involves the ascending aorta, and Stanford type B, which does not involve the ascending aorta [3-5].

Approximately 50% of aortic dissections in women under the age of 40 occur during pregnancy and typically occur in the third trimester or after childbirth [2,3,5]. It corresponds to approximately 0.2% of all aortic dissections and occurs in 0.0004% of pregnancies [1,6]. The physiological changes that take place during pregnancy can place additional stress on the walls of the aorta [3,6,7]. By the 32nd week of gestation, there is a notable increase in intravascular volume, which heightens the risk for dissection at this stage [1,4,6]. Furthermore, estrogen has been found to weaken the structural integrity of the aortic wall, making pregnant individuals more susceptible to dissection [1,6,8]. Additionally, aortocaval compression during the later stages of pregnancy can lead to an increase in afterload and potentially contribute to this condition [1,4,6].

## Case presentation

A 39-year-old Caucasian woman, with an uncomplicated pregnancy of 37 weeks, presented to the emergency department (ED) following an episode of sudden and severe onset of dyspnea and nausea that lasted for a brief period followed by a syncope episode. She had a history of chronic hypertension and was medicated with nifedipine with blood pressure control before and during pregnancy. She had no other medical, surgical, or drug-use history. No seizures or other signs of preeclampsia have been documented.

**Figure 1:**
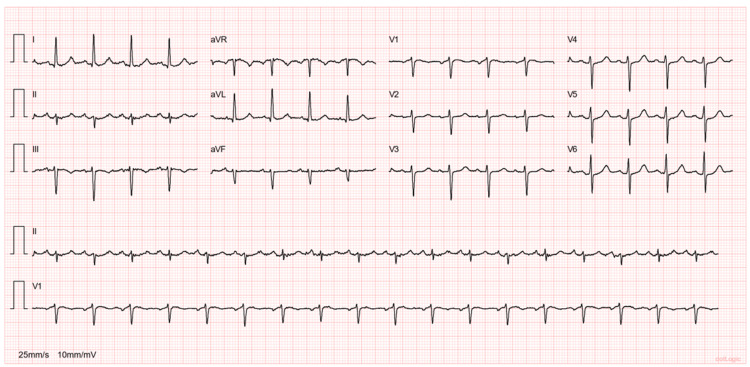
Electrocardiogram An electrocardiogram obtained on arrival shows sinus tachycardia, a left axis deviation, poor R-wave progression in leads V1 through V4, and an electrical alternans (more easily seen in leads III and V1).

At presentation, she felt tired but was conscious and had no other complaints. On physical exam, she was pale. Her heart rate was 110 beats per minute, blood pressure 89/67 mmHg and respiratory rate 20 breaths per minute, and she was saturating at 98% on room air. The patient did not exhibit any signs of Marfan syndrome or other dysmorphic features. She did not have a fever, and there were no indications of lung or peripheral congestion nor any observable bleeding. An electrocardiogram was performed, showing sinus tachycardia, left axis deviation, poor progression of R waves, and electrical alternans (Figure 1).

In light of her critical condition, an immediate cardiology assessment was requested. A bedside ultrasound examination showed an acute Stanford type A aortic dissection accompanied by pericardial effusion that progressed to tamponade (Video 1).

**Video 1:** Transthoracic echocardiography evaluation at the bedside A transthoracic echocardiography was performed at the time of the current presentation while the patient was still communicating with the medical team. In the upper region of the image, it is possible to observe pericardial effusion with right chambers collapse (cardiac tamponade (a)) associated with a dissection of the aorta in the right region of the image (b).

During the echocardiographic evaluation, the patient quickly became unconscious with pulseless electrical activity (PEA) and was swiftly transported to the emergency room. A subxiphoid blind pericardiocentesis approach was performed with more than 200 mL of blood continuously aspirated. Advanced life support measures were initiated with chest compressions, and a rapid sequence orotracheal intubation was performed. The obstetrical team concurrently performed an emergent perimortem cesarean delivery 12 minutes later. A baby boy was delivered, intubated, and transferred to the neonatal intensive care unit.

The patient continued with PEA even after pericardiocentesis and the cesarean. Unfortunately, due to the irreversible nature of the mother's condition, she was pronounced dead approximately one hour after the initial observation. The baby boy was discharged a month later to the care of his father without neurological sequelae.

## Discussion

The occurrence of aortic dissection is typically linked to various risk factors and is uncommon among women under the age of 40 and even rarer in pregnant women [2,3,5]. Certain risk factors like connective tissue disorders (such as Ehlers-Danlos and Marfan syndromes), abnormalities in the aortic valve, enlarged aortic root diameters, coarctation, and high blood pressure can make pregnant patients more susceptible to experiencing dissection earlier in their pregnancy [3,6,9].

Symptoms of aortic dissection include features such as sudden-onset chest pain with radiation to the back [1,3]. However, additional non-specific symptoms like syncope, nausea, vomiting, sweating, and even bronchospasm may occur due to irritation of the vagal nerve [3,10]. Pregnant patients often experience less specific symptoms that are frequently overlooked or underestimated [6,7,11].

An electrocardiogram may be normal or show signs of left ventricular hypertrophy and signs of an acute myocardial infarction if the coronary arteries are involved [4,5,7]. A transesophageal echocardiography (TEE) is considered the most reliable method for diagnosing aortic aneurysm or dissection, especially in a critically ill pregnant patient [12,13]. Nonetheless, in the emergency department where patients are often critical, bedside ultrasound provides a readily accessible alternative and has a high specificity for detecting pericardial effusion and it also enables early therapeutic intervention [5,12].

The treatment strategy for a dissection is determined by the severity of the condition, the stage of pregnancy, and the overall well-being of both the mother and baby [6,10,11]. In a pericardial tamponade subsequent to a proximal aortic dissection, as in this case, pericardiocentesis is the initial treatment indicated to relieve external pressure on the heart and regain cardiac output [10,11]. An open thoracotomy is indicated to evacuate clots and resolve pericardial tamponade that cannot be drained via pericardiocentesis [1,3,10].

In cardiopulmonary arrest, it is recommended that pregnant patients beyond 24 weeks gestation undergo emergent cesarean delivery [9,14,15]. If resuscitation efforts do not yield improvement within four minutes, cesarean delivery should be promptly carried out. However, before reaching 24 weeks gestation, there is a high risk of fetal demise without significant improvement in maternal circulation through delivery [6,14,15]. In our case study, the patient did not have an improvement in hemodynamic stability even after successful emergent cesarean delivery and continued resuscitative efforts were unsuccessful.

The majority of cases discussed in academic literature focus on the need for surgical intervention before acute complications arise [2,5,10]. In an emergency department setting, it is crucial to maintain a heightened level of suspicion for this diagnosis as it is one of the leading causes of death during pregnancy and early intervention can greatly increase the chances of successful treatment [2,3]. In such scenarios where an obstetrician is unavailable, it is crucial for emergency physicians to possess the necessary expertise to carry out this procedure. It is common for healthcare professionals to have knowledge gaps and concerns regarding this particular intervention. In the situation described, all the procedures and interventions were performed within one hour of presentation and despite the patient's inability to be revived, her baby was successfully saved as a result.

## Conclusions

In conclusion, aortic aneurysms and dissections in pregnancy are rare but potentially life-threatening conditions that demand prompt recognition and specialized care. Health professionals should be vigilant about the risk factors and symptoms associated with aortic dissection in pregnant women, as early intervention can significantly impact maternal and fetal outcomes. Raising awareness among medical practitioners can contribute to timely diagnosis and optimal management of this complex medical challenge, ensuring the safety and well-being of both mother and child. Interdisciplinary work is essential in this type of scenario.
